# A multi-faceted comparative perspective on elevational beta-diversity: the patterns and their causes

**DOI:** 10.1098/rspb.2021.0343

**Published:** 2021-04-28

**Authors:** Yuanbao Du, Liqing Fan, Zhenghui Xu, Zhixin Wen, Tianlong Cai, Anderson Feijo, Junhua Hu, Fumin Lei, Qisen Yang, Huijie Qiao

**Affiliations:** ^1^ Key Laboratory of Animal Ecology and Conservation Biology, Institute of Zoology, Chinese Academy of Sciences, Beijing, People's Republic of China; ^2^ National Forest Ecosystem Observation & Research Station of Nyingchi Tibet, Institute of Plateau Ecology, Tibet Agriculture & Animal Husbandry University, Nyingchi, Tibet Autonomous Region, People's Republic of China; ^3^ Key Laboratory of Forest Ecology in Tibet Plateau (Tibet Agriculture & Animal Husbandry University), Ministry of Education, Nyingchi, Tibet Autonomous Region, People's Republic of China; ^4^ Key Laboratory of Zoological Systematics and Evolution, Institute of Zoology, Chinese Academy of Sciences, Beijing, People's Republic of China; ^5^ Key Laboratory of Forest Disaster Warning and Control in Yunnan Province, College of Biodiversity Conservation, Southwest Forestry University, Kunming, Yunnan Province, People's Republic of China; ^6^ School of Life Science, Westlake University, Hangzhou, Zhejiang Province, People's Republic of China; ^7^ Chengdu Institute of Biology, Chinese Academy of Sciences, Chengdu, Sichuan Province, People's Republic of China

**Keywords:** beta-diversity decomposing, turnover and nestedness, multi-dimensional dissimilarity, opposite niche-based processes, Qinghai-Tibet plateau, montane system

## Abstract

The observed patterns and underlying mechanisms of elevational beta-diversity have been explored intensively, but multi-dimensional comparative studies remain scarce. Herein, across distinct beta-diversity components, dimensions and species groups, we designed a multi-faceted comparative framework aiming to reveal the general rules in the observed patterns and underlying causes of elevational beta-diversity. We have found that: first, the turnover process dominated altitudinal patterns of species beta-diversity (*β*_sim_ > *β*_sne_), whereas the nestedness process appeared relatively more important for elevational trait dissimilarity (*β*_funcsim_ < *β*_funcsne_); second, the taxonomic turnover was relative higher than its phylogenetic and functional analogues (*β*_sim_ > *β*_phylosim_/*β*_funcsim_), conversely, nestedness-resultant trait dissimilarity tended to be higher than the taxonomic and phylogenetic measures (*β*_funcsne_ > *β*_sne_/*β*_phylosne_); and third, as elevational distance increased, the contradicting dynamics of environmental filtering and limiting similarity have jointly led the elevational patterns of beta-diversity, especially at taxonomic dimension. Based on these findings, we infer that the species turnover among phylogenetic relatives sharing similar functional attributes appears to be the main cause of shaping the altitudinal patterns of multi-dimensional beta-diversity. Owing to the methodological limitation in the randomization approach, currently, it remains extremely challenging to distinguish the influence of the neutral process from the offset between opposing niche-based processes. Despite the complexities and uncertainties during species assembling, with a multi-dimensional comparative perspective, this work offers us several important commonalities of elevational beta-diversity dynamics.

## 1. Background

Encompassing a large number of endemic and threatened species within extremely limited spaces, montane regions are widely recognized as areas of high priority for conservation [1]. Before establishing any conservation measures and management schemes, one first needs to understand the mechanisms underlying community structure. Beta-diversity, which describes ‘the extent of change in community composition among sites' [2], has become increasingly popular for understanding the drivers and maintenance of biodiversity [3,4]. Early beta-diversity studies primarily focused on taxonomic variation in community composition [2,5] and provided important insight into community assembly. However, as taxonomic classification alone cannot account for functional and evolutionary differentiation, mechanistic inferences on these grounds are increasingly questioned [6–8]. Phylogenetical and trait-based measures of beta-diversity, which estimates the phylogenetic and functional distance among communities, provide new perspectives by connecting local ecological and regional evolutionary processes [6,9,10]. Under the assumption of phylogenetic niche conservatism [7], if ecological niches of species group are phylogenetically conserved, the phylogenetic distance among species could be treated as a surrogate of interspecific dissimilarity at multiple niche dimensions [10–12]. However, ecological niches (frequently measured by ecological traits) are not always phylogenetically conserved [13,14] or, if conserved, cannot be fully accounted for with only a phylogeny [15–17]. These complexities demonstrate the importance of integrating taxonomic, phylogenetic and trait-based dimensions to understand the mechanisms determining community composition over space and time [16,18].

**Figure 1.**
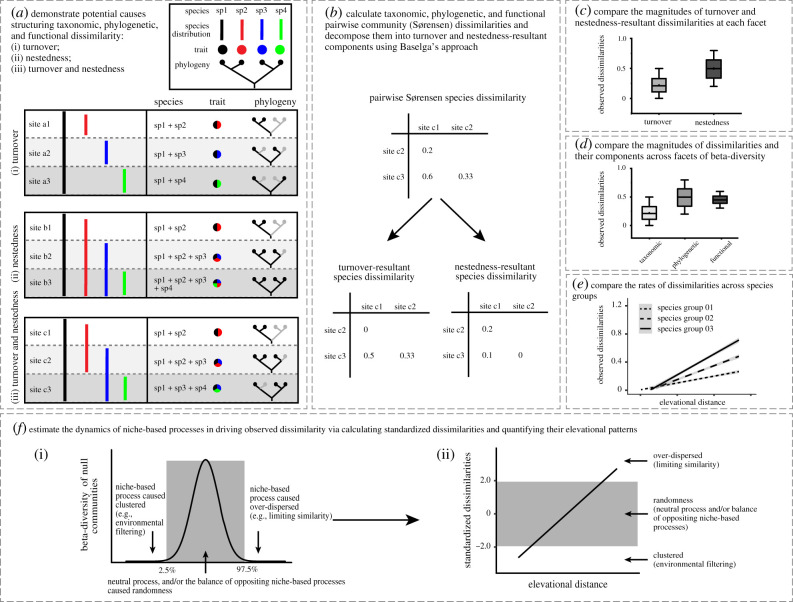
Conceptual figure outlines the analytical approach in this work. First, we demonstrate potential causes structuring taxonomic, phylogenetic and functional dissimilarity (*a*): (i) turnover; (ii) nestedness; and (iii) the joint of turnover and nestedness. Second, we calculate three dimensions of pairwise community dissimilarities and decompose into turnover and nestedness-resultant components following Baselga's approach (*b*). Third, we compare the magnitudes of turnover and nestedness-resultant dissimilarities at each facet of beta-diversity (*c*). Then, we compare the magnitudes of dissimilarities and their components across facets of beta-diversity (*d*). Across three species groups, we compare the rates of increase in dissimilarities (*e*). Lastly, we estimate the dynamics of opposite niche-based processes in driving observed dissimilarity via calculating standardized dissimilarities (*f*) (i) and quantifying their elevational patterns (*f*) (ii). (Online version in colour.)

New approaches for beta-diversity decomposition offer opportunities to understand beta-diversity via the ecological causes of dissimilarity (e.g. spatial turnover and nestedness) [19]. Generally, the turnover component of community dissimilarity represents the contribution of replacement between distinct species, phylogenetic lineages or functional attributes [3] (figure 1*a*i), whereas nestedness-resultant dissimilarity results from ordered extinction or colonization along gradients [20–22] (figure 1*a*ii). Actually, these two antithetical processes are often mixed together (figure 1*a*iii), clearly showing the necessity of beta-diversity decomposing. Several approaches of decomposing community dissimilarity have seen significant study lately [3,23,24], and Baselga's approach based on turnover and nestedness processes is now widely accepted by community ecologists.

The relative importance of niche-based and neutral processes in structuring community has long been debated [25,26]. The neutral hypothesis emphasizes the importance of stochasticity and non-directional processes in driving community assembly [26], whereas the niche-based hypothesis emphasizes interspecific differentiation and non-random responses to environmental gradients [25]. Under the neutral hypothesis, the environment fitness of different organisms is assumed to be equivalent, of which neutral and stochastic processes are expected to be dominant in leading species colonization, spatial distributions, and extinction [27]. That means, under a certain spatial and temporal extent, organisms with more effective dispersal ought to exhibit lower dissimilarity over space or time [28]. However, this prediction is not widely supported by empirical studies [29,30]. Well-known, heterogeneous biotic and abiotic interactions across space and time ought to affect the outcomes of neutral dispersal owing to species mutualisms or antagonisms [31–33]. Currently, the influence of both neutral and deterministic processes is widely accepted [12], but their relative importance and spatial dynamics typically vary across scales, environmental gradients and taxa [17,31–33].

Here, aiming to reveal the general patterns and underlying mechanisms of elevational beta-diversity, we designed a three-level comparative analysis across compositional components (turnover and nestedness), beta-diversity dimensions (species, phylogeny and traits) and taxonomic groups (passerines, rodents and ants) along a Tibetan mountain slope. We decomposed species, phylogenetic and trait-based dissimilarity of three animal groups (figure 1*b*). For the first level of comparison, we examined the magnitude of dissimilarity related to turnover and nestedness processes, and repeated the comparison at each dimension of beta-diversity in the three animal groups (figure 1*c*). For the second level of comparison, aiming to assess the magnitude of dissimilarity across the three beta-diversity dimensions, we performed inter-dimensional comparison on the dissimilarity of turnover, nestedness and their total contributions, respectively (figure 1*d*). Further, we attempted to examine the linear relationships of elevational beta-diversity and their best environmental predictors. If the observed beta-diversity exhibited a significant (*p* ≤ 0.05) linear pattern with elevational distance, we then conducted the third-step of comparison by examining the slopes of linear models across the three animal groups (figure 1*e*). Predictively, we proposed a series of hypotheses (H0) and alternatives (H1) to illustrate potential scenarios and relevant mechanisms in the three-level comparisons (table 1). Lastly, by applying null-model procedures and linear regressions, we examined the dynamics of different niche-based processes (e.g. environmental filtering and negative competition exclusion) in driving elevational beta-diversity (figure 1*f*).

**Table 1.** Potential patterns and relevant mechanisms in the three-level comparisons across components (turnover and nestedness), dimensions (species, phylogeny and traits) and species groups (passerines, rodents and ants). (Non-significant differences could result from the interactions between opposite processes and are not presented.)

comparison levels	hypothesis (H0) and potential mechanisms	alternative hypothesis (H1) and potential mechanisms	reference
1. within dimension	***β*_sim_ > *β*_sne_:** species dissimilarity is driven by species replacement resulting from different environment or strong geographical barrier	***β*_sim_ < *β*_sne_:** species dissimilarity is driven by neutral dispersal and stochastic extinction/colonization	[3]
	***β*_phylosim_ > *β*_phylosne_:** there are strong historical isolation and stable local adaptations for major phylogenetic lineages	***β*_phylosim_ < *β*_phylosne_:** there are frequent historical up-down connections and labile local adaptation	[3,6]
	***β*_funcsim_ > *β*_funcsne_:** stepwised environmental filtering along elevational gradient acts on highly labile functional attributes	***β*_funcsim_ < *β*_funcsne_:** increasingly environmental filtering along elevational gradient acts on the trait of weak lability	[34]
2. across dimensions	***β*_sim_ > *β*_phylosim_/*β*_sne_ > *β*_phylosne_/*β*_sor_ > *β*_phylosor_:** species turnover/loss/gain occurs more likely among phylogenetic relatives	***β*_sim_ < *β*_phylosim_/*β*_sne_ < *β*_phylosne_/*β*_sor_ < *β*_phylosor_:** species turnover/loss/gain frequently happens among distant-related species	[35,36]
	***β*_sim_ > *β*_funcsim_/*β*_sne_ > *β*_funcsne_/*β*_sor_ > *β*_funcsor_:** species turnover/loss/gain frequently occurs among species of similar ecological performance	***β*_sim_ < *β*_funcsim_/*β*_sne_ < *β*_funcsne_/*β*_sor_ < *β*_funcsor_:** species turnover/loss/gain frequently occurs among species with different ecological performance	[35,36]
	***β*_phylosim_ > *β*_funcsim_/*β*_phylosne_ > *β*_funcsne_/*β*_phylosor_ > *β*_funcsor_:** the traits mediating deterministic and stochastic processes are conserved on phylogeny	***β*_phylosim_ < *β*_funcsim_/*β*_phylosne_ < *β*_funcsne_/*β*_phylosor_ < *β*_funcsor_:** the traits mediating deterministic and stochastic processes are convergent on phylogeny	[35,36]
3. across species groups	***β*_ant_ > *β*_rodent_ > *β*_passerine_ (species):** lower dispersal efficiency produces higher beta-diversity; invertebrates are more sensitive to the environmental variations than homothermal mammals and birds	**unpredictable:** different species groups experience different efficient time of dispersal and depend on distinct biotic and abiotic factors; the effect of species pool	[29,32,36]
	***β*_ant_ > *β*_rodent_/*β*_passerine_ (phylogeny):** stronger historical isolation will produce a higher rate of phylogenetic dissimilarity. Historical connections in rodents and passerines are more frequent than ants during the uplift of the Qinghai-Tibet Plateau and glacial-interglacial oscillation	**unpredictable:** different species groups experience distinct evolutionary process at inconsistent temporal scales	[6,37]
	***β*_ant_ > *β*_rodent_/*β*_passerine_ (trait):** higher functional stability and higher environmental persistence produce lower trait beta-diversity	**unpredictable:** local environment adaptations of different species groups rely on a different organism–environment relationship	[38]

## 2. Materials and methods

### (a) Study location and data collection

This work was conducted on the eastern slope of Mt Segrila (29°10′–30°15′ N, 93°12′–95°35′ E) [39] (electronic supplementary material, figure S1), which is located in eastern Nyingchi City, Tibet, China. Located between the Nyainqêntanglha Mountains and the Himalayas, the elevational range of Mt Segrila reaches approximately 3400 m (valley base is approximately 1900 m; mountain summit exceeds 5300 m) [40]. This region has a monsoonal climate, and warm-temperate, needleleaf and broadleaf mixed forests are typical in the low valley zone (1900–2700 m). As elevation increases, the temperate needleleaf forest zone (2700–3300 m), sub-alpine cold-temperate needleleaf forest zone (3300–4200 m), alpine cold-temperate shrub and meadow zone (4200–4500 m) and alpine tundra and desert zone (4500–5300 m) successively dominate on the eastern slope of Mt Segrila [40].

As the primary and secondary consumers and the most important food resources to senior consumers, birds, small mammals and invertebrates, respectively, play important roles in energy transmission and material circulation in various types of biocenoses and ecosystems. Considering our research aims and experimental feasibility, we chose passerines (Passeriformes), rodents (Rodentia) and ants (Formicidae) to represent these fauna groups. Within an approximate 2500 m altitudinal range along the eastern slope of Mt Segrila, we performed field surveys for these three animal groups. The compositional information of passerines was collected from 4 to 10 times' field observations of 17 sampling transects (800 m–2300 m) dating from June 2018/2019 to June 2020. The passerine dataset contained 116 species of 64 genera and 34 families. Field survey on rodents included snap-trapping and field observations. Snap-trappings were conducted from nine sampling sites with two trapping surveys during the early (March–July) and late (July–September) wet seasons in 2014. We also conducted three field observations as snap-trapping, of which the first and second were carried out as snap-trapping and the third field observation was conducted in August 2018. In total, we have collected 14 rodent species, in which two species (*Sciurotamias davidianus* and *Marmota himalayana*) were collected from field observations only. We collected 18 ant species (including three unidentified species) from 11 sampling sites during July–August 2009. As we found no ant in the highest sampling sites (4548 m), they were judged absent there. More detailed descriptions of field collections are given in the appendices (electronic supplementary material, Appendix S01). The datasets of raw presence–absence species composition are available at Dryad (doi:10.5061/dryad.mw6m905wf) [41].

### (b) Phylogeny

Phylogenies of rodents and ants used in phylogenetic beta-diversity analyses were reconstructed using published DNA sequences from GenBank (http://www.ncbi.nlm.nih.gov/genbank/). We inferred the rodent phylogeny using four mitochondrial DNA genes (Cytb, CoI, 12s-rRNA and 16s-rRNA) and three nuclear DNA genes (IRBP, GHR, and RAG1) (electronic supplementary material, figure S2) [15]. To reconstruct the ant phylogeny, we used two mitochondrial DNA genes (CoI and 28s-rRNA). Phylogenetic relationships for ant and rodent species were separately estimated through Bayesian inference using MrBayes (version 3.2.5) [42]. Parameter settings and other detailed information in phylogenetic reconstruction can be found in Du *et al.* [15]. Phylogenetic relationships for passerines were obtained using the online phylogeny tool on the website BirdTree (https://birdtree.org/) [43]. This tool provided a simple way of producing distributions of trees with subsets of bird taxa. This approach followed the same structure and taxonomy as Jetz *et al.* [43], which has been widely used in recent integrative analyses. We chose the default setting ‘Ericson All Species: a set of 10 000 trees with 9993 operational taxonomic units each’ to generate 100 trees that were used in subsequent phylogenetic analyses for passerines. The phylogenetic information for passerines, rodents and ants is available in the electronic supplementary material, Appendix S02.

### (c) Functional traits: size-related morphological attributes

The term ‘functional trait’ is a definition of the measurable function relating to an organism's niche. By measuring the functional aspects of diversity that potentially affect community assembly [44], ‘functional diversity’ describes the extent of functional differences among the species in a community [44–46]. Aiming to conduct comparative analyses across three animal taxa, size-related morphological attributes were used to quantify trait-based beta-diversity. Body size is one of the most important characters affecting animal interspecific competition and resource access [47–49], and size-related morphological characters have been frequently applied in comparative analyses of the functional diversity of the animal community [31,32,50]. For rodents, we characterized size using the mean of each measure (body weight, head-body length, tail length, ear length and hind-foot length) of at least eight adult specimens (four males and four females) for each species [15]. Similarly, six (body weight, body size, bill length, wing length, tail length and plantar) and two (maximum and minimum head-body length of worker-priests) size-related morphological traits were used to represent the size in passerines and ants, respectively. The head-body length of worker-priests indicates the total natural length of the head, thoracic segments and abdominal segments. The measurements were obtained from measuring specimens (rodents), historical records and the literature (passerines and ants). We performed principal component analysis with the transformed (scaled and log-transformed) morphological attributes. As the first two components account for 70–100% of the total variation, they were used to calculate sized-related trait beta-diversity in this work (rodents: electronic supplementary material, figure S2; ants: figure S3). We also assessed the degree of a phylogenetic signal of each size-related morphological attribute using the *K* statistic proposed by Blomberg *et al.* [51]. The measurements of size-related morphologies and phylogenetic signal detection are available in the electronic supplementary material, Appendix S03.

### (d) Environmental variables

In order to assess the dependence of elevational beta-diversity on the local environment, we conducted best predictor selection on five climatic and MODIS factors: annual mean temperature (AMT), annual precipitation (AP), annual mean humidity (AMH), net primary production (NPP) and potential evapotranspiration (PET). AMT, AP and AMH were extracted from the climate records of eight auto local weather stations (Pailong, Nichi, Bingzhan, Lulang, 114zhan, Shengtaizhan, 113zhan, and Shanding) (dating from January 2007 to February 2008) on Mt Segrila (data shown in the electronic supplementary material, Appendix S04) [52]. The three climatic factors were estimated for each sampling site using a linear estimation based on the records of the two nearest weather stations. NPP and PET were extracted from high resolution (1 * 1 km) MODIS products accessed from the office website of Land Processes Distributed Active Archive Center (https://lpdaac.usgs.gov/) (accessed in July 2016) [53,54]. A layer mosaic was performed in ENVI (v. 4.7) [55]. Projection transformation and data extraction were carried out with ArcGIS (v. 10.0) [56].

### (e) Observed and standardized beta-diversities

In this work, the terms ‘community’ or ‘assemblage’ mean focal species occurring in each sampling site. The observed and standardized taxonomic, phylogenetic and trait beta-diversity across communities were calculated based on raw presence–absence community data using the incidence-based pairwise Sørensen dissimilarity index [38] and its phylogenetic and trait-based analogues [9,34]. Trait beta-diversity measures were calculated using the convex hull approach proposed by Villéger *et al*. [34]. Using an additive decomposing approach [3], pairwise beta-diversities were decomposed into turnover and nestedness-resultant dissimilarity components. Under a null model, the standardized measures were calculated for all observed beta-diversities. Owing to the methodological limitation of the convex hull approach, observed and standardized trait beta-diversity measures as well as the associated analyses were only calculated and assessed for assemblages containing three or more species. Detailed information for calculating observed and standardized beta-diversities are provided in the electronic supplementary material, Appendix S05.

### (f) Statistical analyses

The comparisons between turnover and nestedness-resultant dissimilarities, and the dissimilarities across three dimensions of beta-diversity were examined using a two-sided Wilcoxon rank-sum test [57]. The elevational patterns of observed dissimilarity and their components along elevational distances were determined via linear regressions. The Wilcoxon rank-sum test and linear fitting model for trait beta-diversities were only assessed among subset assemblages containing three or more species. Across species groups, we compared the rate of increase in observed dissimilarity with elevational distance by examining the slopes of linear regressions (if it is significant, *p* ≤ 0.05). As the quantification of trait beta-diversity was discordant with each other, the comparisons across animal groups mainly focused on species and phylogenetic dimensions. According to the least Akaike information criterion, the selections of the best predictive variables for each observed beta-diversity measure were performed using a forward selection procedure. Using linear regressions, we examined the elevational patterns of standardized beta-diversity, with the aim to assess the dynamics of different deterministic processes as elevational distance increased.

All statistical analyses were performed in R (v. 3.5.3) [58] using the packages ‘leaps’ [59], ‘foreach’ [60], ‘fBasics’ [61], ‘ape’ [62], ‘ecodist’ [63], ‘picante’ [64], ‘FD’ [65], ‘vegan’ [66] and ‘betapart’ [67]. Box plots and scatter plots were generated using ‘ggplot2’ [68]. The silhouette images of passerine, rodent and ant were freely obtained from PhyloPic (http://phylopic.org/).

## 3. Results

### (a) Three-level's comparison on observed beta-diversity

#### (i) Comparisons between turnover and nestedness processes

**Figure 2.**
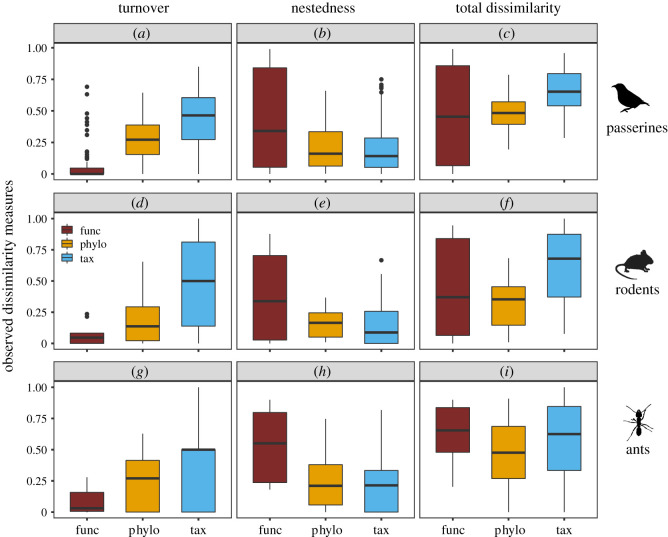
The dissimilarities driven by turnover (*a*, *d* and *g*), nestedness (*b*, *e* and *h*) and their combinations (*c*, *f* and *i*) at taxonomic (‘tax’, light blue (light grey)), phylogenetic (‘phylo’, orange (grey)) and functional (‘func’, dark red (dark grey)) dimensions in passerines (*a*–*c*), rodents (*d*–*f*) and ants (*g*–*i*). Observed functional beta-diversity measures of rodents and ants were calculated using subset assemblages containing three or more species. (Online version in colour.)

According to the results of the Wilcoxon test, the dissimilarity of species turnover was relatively higher than that of nestedness (*β*_sim_ > *β*_sne_), and this was consistent across the three animal groups (passerines: |z| = 6.411, *p* < 0.001; rodents: |z| = 3.243, *p* = 0.001; ants: |z| = 2.382, *p* = 0.017). On the contrary, at trait dimension, the nestedness-resultant dissimilarity was significantly (*p* ≤ 0.05) or nearly significantly (0.05 < *p* ≤ 0.1) higher than that of turnover (passerines: |z| = 8.268, *p* < 0.001; rodents: |z| = 2.857, *p* = 0.001; ants: |z| = 1.887, *p* = 0.059). The comparisons at phylogenetic dimension were inconsistent across the three animal groups (figure 2; electronic supplementary material, table S1).

#### (ii) Comparisons across diversity dimensions

The comparisons of turnover (*β*_sim_, *β*_phylosim_, and *β*_funcsim_) and nestedness-resultant dissimilarity (*β*_sne_, *β*_phylosne_ and *β*_funcsne_) across beta-diversity dimensions exhibited consistent patterns across the three animal groups: species turnover was relatively higher than their phylogenetic and trait analogues (*β*_sim_ > *β*_phylosim_/*β*_funcsim_), whereas nestedness-resultant dissimilarities at trait dimension tended to be higher than their species and phylogenetic analogues (*β*_funcsne_ > *β*_phylosne_/*β*_sne_) (figure 2; electronic supplementary material, table S1). Owing to the uneven joint effects of turnover and nestedness processes, the results of comparative analyses on total dissimilarities were inconsistent across the three species groups (figure 2; electronic supplementary material, table S1).

#### (iii) Comparisons across species groups: the rate of the increase in species and phylogenetic dissimilarity with increasing elevational distance

Across animal groups, we mainly focused on the comparisons at species and phylogenetic dimensions. According to the results of linear regressions, *β*_sim_, *β*_sor_ and *β*_phylosor_ of the three animal groups consistently exhibited significant (*p* < 0.05) monotonic linear patterns, but not for *β*_sne_ of passerines, *β*_sne_ and *β*_phylosne_ of rodents, and *β*_phylosim_ and *β*_sne_ of ants (electronic supplementary material, figure S4 and table S2). By comparing the slopes (*S*) of significant linear fitting models, we found that species turnover and total species dissimilarity of rodents (*S*-*β*_sim_ = 4.66 × 10^−04^ and *S*-*β*_sor_ = 4.02 × 10^−04^) increased more quickly than those of passerines (*S*-*β*_sim_ = 2.05 × 10^−04^ and *S*-*β*_sor_ = 2.00 × 10^−04^) and ants (*S*-*β*_sim_ = 2.00 × 10^−04^ and *S*-*β*_sor_ = 2.50 × 10^−04^). At the phylogenetic dimension, the rate of increase in the phylogenetic dissimilarity of ants (*S*-*β*_phylosor_ = 2.33 × 10^−04^) was slightly higher than that of rodents (*S*-*β*_phylosor_ = 2.08 × 10^−04^) and passerines (*S*-*β*_phylosor_ = 1.50 × 10^−04^) (electronic supplementary material, figure S4 and table S2).

### (b) Best environmental predictors

According to the results of forward model selections for observed measures of beta-diversity, the best environmental predictors were inconsistent across beta-diversity components (turnover and nestedness), dimensions (species, phylogeny and trait) and animal groups (passerines, rodents and ants) (electronic supplementary material, table S3). Among five environmental predictors, NPP significantly contributed to the elevational patterns of 17 observed beta-diversity measures, followed by MAT (14), PET (10) and AP (six). By comparison, AMH significantly contributed to only three observed beta-diversity measures (electronic supplementary material, table S3).

### (c) Standard pairwised beta-diversity measures: the dynamics of opposite deterministic processes

**Figure 3.**
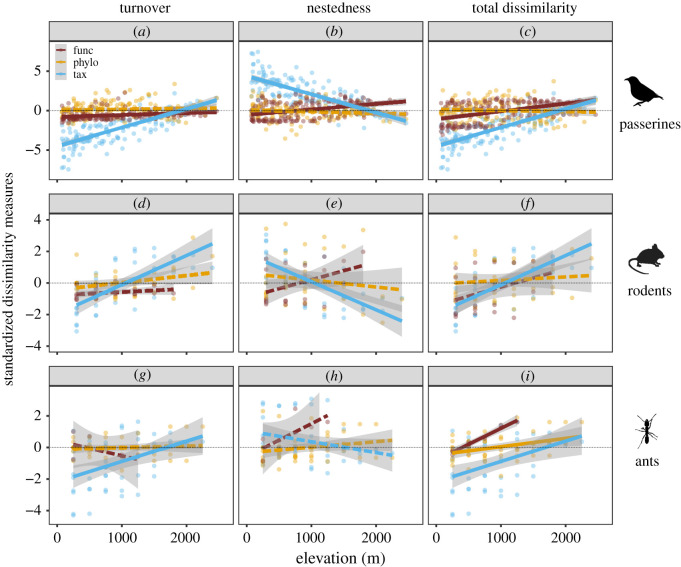
Linear elevational patterns of standardized dissimilarities driven by turnover (*a*, *d* and *g*), nestedness (*b*, *e* and *h*) and their combinations (*c*, *f* and *i*) at taxonomic (‘tax’, light blue (light grey)), phylogenetic (‘phylo’, orange (grey)) and functional (‘func’, dark red (dark grey)) dimensions in passerines (*a–c*), rodents (*d–f*) and ants (*g–i*). Solid lines indicate that linear fittings are significant at the level of *p* ≤ 0.05, whereas dash lines imply that linear fittings are non-significant (*p* > 0.05). The standardized functional beta-diversity measures of rodents and ants were calculated using subset assemblages containing three or more species. (Online version in colour.)

According to the results of the linear regressions, the standardized taxonomic measures (SES . *β*_sim_, SES . *β*_sne_ and SES . *β*_sor_) of the three animal groups consistently exhibited significant (*p* ≤ 0.05) or nearly significant (0.05 < *p* ≤ 0.1) linear patterns as elevational distance increased. Most of the standardized phylogenetic and trait beta-diversity measures of three animal groups exhibited non-significant linear patterns along the elevational gradient, except for SES . *β*_funcsim_ and SES . *β*_funcsor_ of passerines (*p* = 0.003 and *p* < 0.001) and SES . *β*_phylosor_ of ants (*p* = 0.014) (figure 3 and electronic supplementary material, table S4).

## 4. Discussion

Revealing the general rules of species coexistence and the underlying processes thereof are common goals of community ecologists. With such an ambition, by comparing the observed patterns (the dissimilarity and the rate of increase in dissimilarity) and the dynamics of niche-based drivers of beta-diversity, we present a stepwise (beta-diversity components, dimensions and species groups) comparative framework for revealing the consistence in the patterns and underlying mechanism of elevational beta-diversity.

Spatial turnover and nestedness are recognized as two antithetic processes leading to community compositional variance along environmental gradients [3]. However, their relative importance often depends on the focal taxon and diversity dimension of interest [21,35,69,70]. Generally, species beta-diversity can be indicative of the species' response to the current environment and/or geographical barrier, whereas trait beta-diversity refers to the taxon-specialized adaptation to the varying environment [35]. The comparative results in three animal groups suggest that species replacement and functional loss/gain may happen similarly in different animal groups responding to the varying environment along an elevational gradient. This is especially true when habitat endemics account for a large proportion of the regional species pool. This contrasting pattern in species and functional beta-diversity has been previously reported in European fish assemblages [34] and South African ant communities [21], implying this pattern is common across ecosystems and taxa. Nevertheless, it is worth noting that the patterns of functional beta-diversity are definitely dependent on the subjective bias (e.g. the selections on trait and diversity metric) and taxon-specialized functional character. Hence, the commonness of the dominion of nestedness in driving functional beta-diversity requires further examinations in various ecosystems.

At the phylogenetic dimension, the comparison between turnover and nestedness-resultant dissimilarity in three animal groups reveals inconsistent patterns, disagreeing with the inference that phylogenetic beta-diversity is dominated by phylogenetic turnover [69,71]. Given that phylogenetic beta-diversity indicates the degree of historical isolation across assemblages at an evolutionary timescale [6,35], the inconsistency at the phylogenetic dimension is understandable. On the one hand, phylogenetic beta-diversity highly depends on the taxon-specialized ecological and evolutionary characteristics (e.g. species pool and phylogenetic scale) [6,7], which explicitly differ across the animal groups involved in this work. On the other hand, the regional evolutionary history has probably left distinct evolutionary imprints on different fauna groups. For instance, the uplift of Qinghai-Tibet Plateau and the glacial-interglacial oscillations have different impacts on homeothermic vertebrates and heterothermic invertebrates via distinct magnitudes of preventing or promoting up-down species exchange [37,72,73]. The accumulation of these complexities might explain the inconsistent patterns of elevational phylogenetic beta-diversity in different animal groups.

The comparisons of beta-diversity across species, phylogenetic and trait dimensions may shed additional light on the processes organizing species [36]. We found that turnover-resultant species dissimilarities in the three species groups are consistently higher than their phylogenetic and functional analogues (*β*_sim_ > *β*_phylosim_/*β*_funcsim_), whereas functional nestedness-resultant dissimilarities are consistently higher than their species and phylogenetic analogues (*β*_funcsne_ > *β*_phylosne_/*β*_sne_). These results imply that the assembling processes have produced different but interdependent outcomes at distinct beta-diversity dimensions. As the major force in structuring elevational beta-diversity at taxonomic dimension, species replacement often occurs between phylogenetic relatives [6,9,36,74] sharing similar functional attributes [34,36], which has produced a high level of functional gain or loss. This evidence reinforces the necessity of integrating multi-dimensional information in estimating assembly processes.

Under the same spatio-temporal extent, higher rates of variation in taxonomic composition suggest higher levels of sensitivity to the changing environment and/or stronger dispersal limitation for the focal species group; higher rates of phylogenetic variations indicate stronger historical isolation, and higher rates of trait variations suggest stronger trait specificity limiting adaptation to different environments [34,35]. At the taxonomic dimension, considering environmental persistence, environmental sensitivity and dispersal ability, the taxonomic dissimilarity of heterothermic invertebrates, such as ants, is as expected to be more sensitive to environmental change than that of homeothermic vertebrates (e.g. passerines and rodents). Unexpectedly, both species turnover and total species dissimilarity in rodents, not ants, displayed higher rates of increase of taxonomic dissimilarity with elevational distance. Occupying different niche positions, passerines, rodents and ants are expected to be sensitive to the variation of distinct environmental factors along the elevational gradient [9,30] (also supported in our environment dependence analyses). The higher rates of variations in rodent species composition could result from their sensitivity to the biotic and abiotic conditions of their microhabitat (i.e. food resource) [75,76]. Alternatively, the higher rate of increase of species beta-diversity of rodents could be attributed to the effects of species pool and taxonomic scale, which admittedly could affect the patterns of beta-diversity [77–79]. At the phylogenetic dimension, as mentioned before, the distinct evolutionary histories and the inconsistent effect of regional evolutionary events have jointly shaped the taxon-specialized phylogenetic beta-diversity. At increasing elevational distance, the higher rate of increase in the phylogenetic dissimilarity of ants probably results from severer evolutionary isolation and more stable habitat adaptation.

The results of environment dependence analyses reveal the importance of non-random species-environment interactions in affecting species assembly along an elevational gradient. Although the best environmental predictors varied according to the beta-diversity component, diversity dimension and species group, NPP and AMT had the most widespread effect in explaining the elevational patterns of multiple beta-diversity measures. These results indirectly reflect the importance of the primary producer (providing food and habitat) and physiological limitation in organizing animal assemblages. By comparison, owing to the localized monsoon climate and relative high altitude in our study area, AP appears relatively less important for the observed beta-diversity of the three animal groups. Although PET is an index for energy availability similar to temperature sum, it appears less as an interpretation for elevational beta-diversity owing to its low-data quality (extracted from a global data layer with resolution of 1 km^2^). As an air index weakly related to animal life history, it is understandable that AMH displays a weak role in predicting the elevational patterns of animal beta-diversity in this study.

Aiming to assess the dynamics of opposite niche-based processes, we examined the elevational pattern of standardized beta-diversity via linear regressions. Although there was a relatively higher proportion of beta-diversity classified as random, we cannot fully assert that neutral processes are the dominant or only drivers of beta-diversity along this elevational gradient because the linear fitting models of standardized beta-diversity along an elevational gradient show that deterministic processes dynamically transformed with increasing elevational distance, especially for the elevational beta-diversity at taxonomic dimension. The elevational patterns of standardized species turnover reveal that, as elevational distance increased, the force of environmental filtering consistently decreased (e.g. similar environmental filtering across assemblages), while the magnitude of limiting similarity (e.g. negative interspecific competition) gradually increased. Conversely, the decreasing limiting similarity and the increasing environmental filtering jointly contributed to the elevational patterns of nestedness-resultant dissimilarity. It is worth noting that the strength of environmental filtering and negative interspecific effect became balanced in the median elevational distance. In other words, the high proportion of beta-diversity categorized as random could result from the balance between opposite deterministic processes as well as stochasticity. Despite intensive efforts of ecologists, currently it remains an extraordinary challenge to fully disentangle the effects of opposite deterministic processes (i.e. environmental filtering and interspecific competition) in driving beta-diversity. This methodological flaw of the randomization approach is an urgent issue that needs to be resolved in the future. Nonetheless, this work has represented a substantial improvement to our current knowledge of the general mechanisms underlying multi-dimensional elevational beta-diversity.

## 5. Conclusion

With extensive empirical evidence across three representative animal groups, this work provides a synthetic perspective to understand the underlying processes driving multi-dimensional elevational beta-diversity. We performed a multi-faceted comparative analysis across beta-diversity compositional components (turnover and nestedness), multiple dimensions (species, phylogeny and trait) and species groups (passerines, rodents and ants). The three animal groups were generally consistent on three main points: first, a turnover process dominated the species beta-diversity along an elevational gradient, whereas nestedness process was the main cause for trait-based dissimilarity; second, in the comparison across beta-diversity dimensions, species turnover appeared gradually higher than its phylogenetic and trait-based measures (*β*_sim_ > *β*_phylosim_/*β*_funcsim_); conversely, functional nestedness was relative higher than its taxonomic and phylogenetic analogues (*β*_funcsne_ > *β*_sne_/*β*_phylosne_); and third, the relative importance of opposite niche-based processes (environmental filtering and negative competitive exclusion) gradually transformed in driving elevational beta-diversity as the elevational distance increases. Further, via a linear fitting model and forward selection procedure, we found that the elevational beta-diversity patterns and their best environmental predictors both varied across beta-diversity components, dimensions and species groups. Among five environmental factors, NPP and AMT generally performed stronger effects in explaining the elevational patterns of multiple beta-diversity measures. In the comparisons across species groups, rodents were found to display a higher rate of variation in taxonomic dissimilarity with elevational distance, whereas ants exhibited a higher rate of variation in phylogenetic relatedness. Although we have authenticated the dynamics of opposite niche-based processes, currently it is an extraordinary challenge to disentangle the effects of neutral process and the balance of opposing niche-based processes. Despite the complexities and uncertainties in the ecological and evolutionary processes, this study sets a foundation for bettering our understanding of elevational beta-diversity dynamics.

## Data accessibility

All data referred within this study are freely available as follows: sampling information and species composition are available from the Dryad Digital Repository: https://doi.org/10.5061/dryad.mw6m905wf [41]; detailed information of data collection: electronic supplementary material, Appendix S01; phylogeneies: electronic supplementary material, Appendix S02; trait attributes and phylogenetic signal detection: electronic supplementary material, Appendix S03; information of eight auto weather stations: electronic supplementary material, Appendix S04; information for calculating observed and standardized beta-diversities: electronic supplementary material, Appendix S05; electronic supplementary material, figures and tables: Appendix S06.

## References

[1] Myers N, Mittermeier RA, Mittermeier CG, da Fonseca GA, Kent J. 2000 Biodiversity hotspots for conservation priorities. Nature **403**, 853-858. (10.1038/35002501) 10706275

[2] Whittaker RH. 1960 Vegetation of the Siskiyou Mountains, Oregon and California. Ecol. Monogr. **30**, 280-338. (10.2307/1943563)

[3] Baselga A. 2010 Partitioning the turnover and nestedness components of beta diversity. Glob. Ecol. Biogeogr. **19**, 134-143. (10.1111/j.1466-8238.2009.00490.x)

[4] Condit R et al. 2002 Beta-diversity in tropical forest trees. Science **295**, 666-669. (10.1126/science.1066854) 11809969

[5] Koleff P, Gaston KJ, Lennon JJ. 2003 Measuring beta diversity for presence-absence data. J. Anim. Ecol. **72**, 367-382. (10.1046/j.1365-2656.2003.00710.x)

[6] Graham CH, Fine PV. 2008 Phylogenetic beta diversity: linking ecological and evolutionary processes across space in time. Ecol. Lett. **11**, 1265-1277. (10.1111/j.1461-0248.2008.01256.x) 19046358

[7] Wiens JJ, Graham CH. 2005 Niche conservatism: integrating evolution, ecology, and conservation biology. Annu. Rev. Ecol. Evol. Syst. **36**, 519-539. (10.1146/annurev.ecolsys.36.102803.095431)

[8] Losos JB. 2008 Phylogenetic niche conservatism, phylogenetic signal and the relationship between phylogenetic relatedness and ecological similarity among species. Ecol. Lett. **11**, 995-1003. (10.1111/j.1461-0248.2008.01229.x) 18673385

[9] Bryant JA, Lamanna C, Morlon H, Kerkhoff AJ, Enquist BJ, Green JL. 2008 Colloquium paper: microbes on mountainsides: contrasting elevational patterns of bacterial and plant diversity. Proc. Natl Acad. Sci. USA **105**(Suppl 1), 11 505-11 511. (10.1073/pnas.0801920105) 18695215 PMC2556412

[10] Graham CH, Parra JL, Rahbek C, McGuire JA. 2009 Phylogenetic structure in tropical hummingbird communities. Proc. Natl Acad. Sci. USA **106**(Suppl 2), 19 673-19 678. (10.1073/pnas.0901649106) PMC2780942 19805042

[11] Swenson NG. 2013 The assembly of tropical tree communities the advances and shortcomings of phylogenetic and functional trait analyses. Ecography **36**, 264-276. (10.1111/j.1600-0587.2012.00121.x)

[12] Zhang JL, Swenson NG, Chen SB, Liu XJ, Li ZS, Huang JH, Mi XC, Ma KP. 2013 Phylogenetic beta diversity in tropical forests: implications for the roles of geographical and environmental distance. J. Syst. Evol. **51**, 71-85. (10.1111/j.1759-6831.2012.00220.x)

[13] Cavender-Bares J, Ackerly DD, Baum DA, Bazzaz FA. 2004 Phylogenetic overdispersion in Floridian oak communities. Am. Nat. **163**, 823-843. (10.1086/386375) 15266381

[14] Losos JB, Leal M, Glor RE, De Queiroz K, Hertz PE, Rodriguez Schettino L, Lara AC, Jackman TR, Larson A. 2003 Niche lability in the evolution of a Caribbean lizard community. Nature **424**, 542-545. (10.1038/nature01814) 12891355

[15] Du Y, Wen Z, Zhang J, Lv X, Cheng J, Ge D, Xia L, Yang Q. 2017 The roles of environment, space, and phylogeny in determining functional dispersion of rodents (Rodentia) in the Hengduan Mountains, China. Ecol. Evol. **7**, 10 941-10 951. (10.1002/ece3.3613) PMC5743695 29299271

[16] Swenson NG, Enquist BJ. 2009 Opposing assembly mechanisms in a neotropical dry forest: implications for phylogenetic and functional community ecology. Ecology **90**, 2161-2170. (10.1890/08-1025.1) 19739378

[17] Yang J et al. 2014 Functional and phylogenetic assembly in a Chinese tropical tree community across size classes, spatial scales and habitats. Funct. Ecol. **28**, 520-529. (10.1111/1365-2435.12176)

[18] Cavender-Bares J, Kozak KH, Fine PV, Kembel SW. 2009 The merging of community ecology and phylogenetic biology. Ecol. Lett. **12**, 693-715. (10.1111/j.1461-0248.2009.01314.x) 19473217

[19] Baselga A, Leprieur F. 2015 Comparing methods to separate components of beta diversity. Methods Ecol. Evol. **6**, 1069-1079. (10.1111/2041-210x.12388)

[20] Baselga A. 2012 The relationship between species replacement, dissimilarity derived from nestedness, and nestedness. Glob. Ecol. Biogeogr. **21**, 1223-1232. (10.1111/j.1466-8238.2011.00756.x)

[21] Bishop TR, Robertson MP, van Rensburg BJ, Parr CL. 2015 Contrasting species and functional beta diversity in montane ant assemblages. J. Biogeogr. **42**, 1776-1786. (10.1111/jbi.12537) 27563167 PMC4979679

[22] Ulrich W, Almeida M, Gotelli NJ. 2009 A consumer's guide to nestedness analysis. Oikos **118**, 3-17. (10.1111/j.1600-0706.2008.17053.x)

[23] Carvalho JC, Cardoso P, Gomes P. 2012 Determining the relative roles of species replacement and species richness differences in generating beta-diversity patterns. Glob. Ecol. Biogeogr. **21**, 760-771. (10.1111/j.1466-8238.2011.00694.x)

[24] Podani J, Schmera D. 2011 A new conceptual and methodological framework for exploring and explaining pattern in presence-absence data. Oikos **120**, 1625-1638. (10.1111/j.1600-0706.2011.19451.x)

[25] Chase JM, Leibold MA. 2003 Ecological niches: linking classical and contemporary approaches. Chicago, IL: University of Chicago Press.

[26] Hubbell S. 2001 A unified theory of biodiversity and biogeography. Princeton, NJ: Princeton University Press.

[27] Rafajlovic M et al. 2017 Neutral processes forming large clones during colonization of new areas. J. Evol. Biol. **30**, 1544-1560. (10.1111/jeb.13124) 28557006

[28] Dobrovolski R, Melo AS, Cassemiro FAS, Diniz JAF. 2012 Climatic history and dispersal ability explain the relative importance of turnover and nestedness components of beta diversity. Glob. Ecol. Biogeogr. **21**, 191-197. (10.1111/j.1466-8238.2011.00671.x)

[29] Harrison S, Ross SJ, Lawton JH. 1992 Beta-diversity on geographic gradients in Britain. J. Anim. Ecol. **61**, 151-158. (10.2307/5518).

[30] Zellweger F, Roth T, Bugmann H, Bollmann K. 2017 Beta diversity of plants, birds and butterflies is closely associated with climate and habitat structure. Glob. Ecol. Biogeogr. **26**, 898-906. (10.1111/geb.12598)

[31] Soininen J, Heino J, Wang JJ. 2018 A meta-analysis of nestedness and turnover components of beta diversity across organisms and ecosystems. Glob. Ecol. Biogeogr. **27**, 96-109. (10.1111/geb.12660)

[32] Soininen J, Lennon JJ, Hillebrand H. 2007 A multivariate analysis of beta diversity across organisms and environments. Ecology **88**, 2830-2838. (10.1890/06-1730.1) 18051652

[33] Wang JJ, Soininen J, Zhang Y, Wang BX, Yang XD, Shen J. 2012 Patterns of elevational beta diversity in micro- and macroorganisms. Glob. Ecol. Biogeogr. **21**, 743-750. (10.1111/j.1466-8238.2011.00718.x)

[34] Villéger S, Grenouillet G, Brosse S. 2013 Decomposing functional β-diversity reveals that low functional β-diversity is driven by low functional turnover in European fish assemblages. Glob. Ecol. Biogeogr. **22**, 671-681. (10.1111/geb.12021)

[35] Weinstein BG, Tinoco B, Parra JL, Brown LM, McGuire JA, Stiles FG, Graham CH. 2014 Taxonomic, phylogenetic, and trait beta diversity in South American hummingbirds. Am. Nat. **184**, 211-224. (10.1086/676991) 25058281

[36] Siefert A, Ravenscroft C, Weiser MD, Swenson NG. 2013 Functional beta-diversity patterns reveal deterministic community assembly processes in eastern North American trees. Glob. Ecol. Biogeogr. **22**, 682-691. (10.1111/geb.12030)

[37] Lei F, Qu Y, Song G, Alstrom P, Fjeldsa J. 2015 The potential drivers in forming avian biodiversity hotspots in the East Himalaya Mountains of Southwest China. Integr. Zool. **10**, 171-181. (10.1111/1749-4877.12121) 25316284

[38] Sørensen T. 1948 A method of establishing groups of equal amplitude in plant sociology based on similarity of species and its application to analyses of the vegetation on Danish commons, vol. 5, pp. 1-34. Copenhagen, Denmark: Munksgaard.

[39] Gai JP, Tian H, Yang FY, Christie P, Li XL, Klironomos JN. 2012 Arbuscular mycorrhizal fungal diversity along a Tibetan elevation gradient. Pedobiologia **55**, 145-151. (10.1016/j.pedobi.2011.12.004)

[40] Yong C, Fan GS, Xiang-Wang LI, Zheng WL. 2004 Study on vertical distributional belts and their floristic characters of seed plants from Shegyla Mountains of Xizang (Tibet). China. Guangxi Zhiwu **24**, 107-112. 106.

[41] Du Y. 2021 Data from: Raw presence–absence community composition of passerines, rodents and ants at eastern slope of Mt Segrila. Dryad Digital Repository. (10.5061/dryad.mw6m905wf)

[42] Ronquist F et al. 2012 MrBayes 3.2: efficient Bayesian phylogenetic inference and model choice across a large model space. Syst. Biol. **61**, 539-542. (10.1093/sysbio/sys029) 22357727 PMC3329765

[43] Jetz W, Thomas GH, Joy JB, Hartmann K, Mooers AO. 2012 The global diversity of birds in space and time. Nature **491**, 444-448. (10.1038/nature11631) 23123857

[44] Cadotte MW, Carscadden K, Mirotchnick N. 2011 Beyond species: functional diversity and the maintenance of ecological processes and services. J. Appl. Ecol. **48**, 1079-1087. (10.1111/j.1365-2664.2011.02048.x)

[45] Petchey OL, Gaston KJ. 2002 Functional diversity (FD), species richness and community composition. Ecol. Lett. **5**, 402-411. (10.1046/j.1461-0248.2002.00339.x)

[46] Tilman D. 2001 Functional diversity. Encyclopedia Biodivers. **3**, 109-120. (10.1016/B0-12-226865-2/00132-2)

[47] Blackburn TM, Brown VK, Doube BM, Greenwood JJD, Lawton JH, Stork NE. 1993 The relationship between abundance and body-size in natural animal assemblages. J. Anim. Ecol. **62**, 519-528. (10.2307/5201)

[48] Blackburn TM, Gaston KJ. 1994 Animal body size distributions: patterns, mechanisms and implications. Trends Ecol. Evol. **9**, 471-474. (10.1016/0169-5347(94)90311-5) 21236925

[49] Lafferty KD, Kuris AM. 2002 Trophic strategies, animal diversity and body size. Trends Ecol. Evol. **17**, 507-513. (10.1016/S0169-5347(02)02615-0)

[50] McGill BJ, Enquist BJ, Weiher E, Westoby M. 2006 Rebuilding community ecology from functional traits. Trends Ecol. Evol. **21**, 178-185. (10.1016/j.tree.2006.02.002) 16701083

[51] Blomberg SP, Garland Jr T, Ives AR. 2003 Testing for phylogenetic signal in comparative data: behavioral traits are more labile. Evolution **57**, 717-745. (10.1111/j.0014-3820.2003.tb00285.x) 12778543

[52] Zhuo G, Bianba C, Wang J, Lan X. 2010 Analysis of regional climate characteristics of Tibetan herbal products growing on Mt. Seqilha. J. Resources Sci. **8**, 22–31.

[53] Mu Q, Heinsch FA, Zhao M, Running SW. 2007 Development of a global evapotranspiration algorithm based on MODIS and global meteorology data. Remote Sens. Environ. **111**, 519-536. (10.1016/j.rse.2007.04.015)

[54] Mu QZ, Zhao MS, Running SW. 2011 Improvements to a MODIS global terrestrial evapotranspiration algorithm. Remote Sens. Environ. **115**, 1781-1800. (10.1016/j.rse.2011.02.019)

[55] VIS, I. 2011 ENVI 4.7-The environment for visualizing images. Boulder, CO: ITT Visual Information Solutions.

[56] ESRI. 2010 Arcmap Arclnfo version 10.0. Redlands, CA: Environmental Systems Research Institute, Inc.

[57] Wilcoxon F. 1945 Individual comparisons by ranking methods. Biom. Bullet. **1**, 80-83. (10.2307/3001968)

[58] R Core Team. 2019 *R: a language and environment for statistical computing*. Vienna, Austria: R Foundation for Statistical Computing. See https://www.R-project.org/.

[59] Miller, T.L.b.o.F.c.b.A. 2017 leaps: regression subset selection. See https://CRAN.R-project.org/package=leaps.

[60] Microsoft & Steve, W. 2019 foreach: provides foreach looping construct. R package version 1.4.7. See https://CRAN.R-project.org/package=foreach.

[61] Wuertz D, Setz T, Chalabi Y. 2017 fBasics: rmetrics - markets and basic statistics. R package version 3042.89. See https://CRAN.R-project.org/package=fBasics/.

[62] Paradis E, Schliep K. 2018 ape 5.0: an environment for modern phylogenetics and evolutionary analyses in R. Bioinformatics **35**, 526-528. (10.1093/bioinformatics/bty633) 30016406

[63] Goslee SC, Urban DL. 2007 The ecodist package for dissimilarity-based analysis of ecological data. J. Stat. Softw. **22**, 1-19. (10.18637/jss.v022.i07)

[64] Kembel SW, Cowan PD, Helmus MR, Cornwell WK, Morlon H, Ackerly DD, Blomberg SP, Webb CO. 2010 Picante: R tools for integrating phylogenies and ecology. Bioinformatics **26**, 1463-1464. (10.1093/bioinformatics/btq166) 20395285

[65] Laliberte E, Legendre P. 2010 A distance-based framework for measuring functional diversity from multiple traits. Ecology **91**, 299-305. (10.1890/08-2244.1) 20380219

[66] Oksanen J et al. 2019 vegan: community ecology package. R package version 2, 5–5. See https://CRAN.R-project.org/package=vegan.

[67] Baselga A, Orme D, Villeger S, De Bortoli J, Leprieur F. 2018 betapart: partitioning beta diversity into turnover and nestedness components. R package version 1.5.1. See https://CRAN.R-project.org/package=betapart.

[68] Wickham H. 2016 *ggplot2: elegant graphics for data analysis*. New York, NY: Springer-Verlag.

[69] Branco CCZ, Bispo PC, Peres CK, Tonetto AF, Krupek RA, Barfield M, Holt RD. 2020 Partitioning multiple facets of beta diversity in a tropical stream macroalgal metacommunity. J. Biogeogr. **47**, 1765-1780. (10.1111/jbi.13879)

[70] Leao-Pires TA, Luiz AM, Sawaya RJ. 2018 The complex roles of space and environment in structuring functional, taxonomic and phylogenetic beta diversity of frogs in the Atlantic Forest. PLoS ONE **13**, e0196066. (10.1371/journal.pone.0196066) 29672575 PMC5908149

[71] Peixoto FP, Braga PHP, Cianciaruso MV, Diniz JAF, Brito D. 2014 Global patterns of phylogenetic beta diversity components in bats. J. Biogeogr. **41**, 762-772. (10.1111/jbi.12241)

[72] Päckert M, Martens J, Sun Y-H, Tietze DT. 2015 Evolutionary history of passerine birds (Aves: Passeriformes) from the Qinghai–Tibetan plateau: from a pre-Quarternary perspective to an integrative biodiversity assessment. J. Ornithol. **156**, 355-365. (10.1007/s10336-015-1185-6)

[73] Lei FM, Qu YH, Song G. 2014 Species diversification and phylogeographical patterns of birds in response to the uplift of the Qinghai-Tibet Plateau and Quaternary glaciations. Curr. Zool. **60**, 149-161. (10.1093/czoolo/60.2.149)

[74] Qian H, Swenson NG, Zhang JL. 2013 Phylogenetic beta diversity of angiosperms in North America. Glob. Ecol. Biogeogr. **22**, 1152-1161. (10.1111/geb.12076)

[75] Thompson SD. 1982 Microhabitat utilization and foraging behavior of bipedal and quadrupedal hetermoyid rodents. Ecology **63**, 1303-1312. (10.2307/1938858)

[76] Lessard JP, Borregaard MK, Fordyce JA, Rahbek C, Weiser MD, Dunn RR, Sanders NJ. 2012 Strong influence of regional species pools on continent-wide structuring of local communities. Proc. R. Soc. B **279**, 266-274. (10.1098/rspb.2011.0552) PMC3223666 21676973

[77] de Bello F et al. 2012 Functional species pool framework to test for biotic effects on community assembly. Ecology **93**, 2263-2273. (10.1890/11-1394.1) 23185887

[78] Graham CH, Storch D, Machac A. 2018 Phylogenetic scale in ecology and evolution. Glob. Ecol. Biogeogr. **27**, 175-187. (10.1111/geb.12686)

[79] Cavender-Bares J, Keen A, Miles B. 2006 Phylogenetic structure of Floridian plant communities depends on taxonomic and spatial scale. Ecology **87**, S109-S122. (10.1890/0012-9658(2006)87[109:psofpc]2.0.co;2) 16922307

